# Intrinsic *MYH7* expression regulation contributes to tissue level allelic imbalance in hypertrophic cardiomyopathy

**DOI:** 10.1007/s10974-017-9486-4

**Published:** 2017-11-03

**Authors:** Judith Montag, Mandy Syring, Julia Rose, Anna-Lena Weber, Pia Ernstberger, Anne-Kathrin Mayer, Edgar Becker, Britta Keyser, Cristobal dos Remedios, Andreas Perrot, Jolanda van der Velden, Antonio Francino, Francesco Navarro-Lopez, Carolyn Yung Ho, Bernhard Brenner, Theresia Kraft

**Affiliations:** 1 Institute of Molecular and Cell Physiology, Hannover Medical School, Hanover, Germany; 2 Institute of Human Genetics, Hannover Medical School, Hanover, Germany; 3 Department of Anatomy, Bosch Institute, University of Sydney, Sydney, Australia; 4 Experimental and Clinical Research Center, Charité-University Clinic Berlin, Berlin, Germany; 5 Department of Physiology, Institute for Cardiovascular Research, VU University, Amsterdam, The Netherlands; 6 Hospital Clinic/IDIBAPS, University of Barcelona, Barcelona, Spain; 7 Brigham and Women’s Hospital, Boston, MA USA

**Keywords:** Hypertrophic cardiomyopathy, Allelic imbalance, MYH7, Beta-myosin

## Abstract

HCM, the most common inherited cardiac disease, is mainly caused by mutations in sarcomeric genes. More than a third of the patients are heterozygous for mutations in the *MYH7* gene encoding for the β-myosin heavy chain. In HCM-patients, expression of the mutant and the wildtype allele can be unequal, thus leading to fractions of mutant and wildtype mRNA and protein which deviate from 1:1. This so-called allelic imbalance was detected in whole tissue samples but also in individual cells. There is evidence that the severity of HCM not only depends on the functional effect of the mutation itself, but also on the fraction of mutant protein in the myocardial tissue. Allelic imbalance has been shown to occur in a broad range of genes. Therefore, we aimed to examine whether the *MYH7*-alleles are intrinsically expressed imbalanced or whether the allelic imbalance is solely associated with the disease. We compared the expression of *MYH7*-alleles in non-HCM donors and in HCM-patients with different *MYH7-*missense mutations. In the HCM-patients, we identified imbalanced as well as equal expression of both alleles. Also at the protein level, allelic imbalance was determined. Most interestingly, we also discovered allelic imbalance and balance in non-HCM donors. Our findings therefore strongly indicate that apart from mutation-specific mechanisms, also non-HCM associated allelic-mRNA expression regulation may account for the allelic imbalance of the *MYH7* gene in HCM-patients. Since the relative amount of mutant mRNA and protein or the extent of allelic imbalance has been associated with the severity of HCM, individual analysis of the *MYH7*-allelic expression may provide valuable information for the prognosis of each patient.

## Introduction

HCM is the most common disease of the heart with an incidence of 1:500 (Maron et al. 2012). Mutations in different sarcomeric genes have been shown to account for most of the HCM-cases (Cecconi et al. 2016; Maron et al. 2012). One of the most affected genes is *MYH7* (Marsiglia and Pereira 2014), encoding for the β-myosin heavy chain (β-MyHC), a central player in cardiac and slow muscle contraction.

Numerous mutations in the *MYH7*-gene have been associated with HCM (Walsh et al. 2009). Since mutations in the *MYH7*-gene are mostly missense mutations, a poison peptide effect has been postulated for the mutant proteins. With respect to disease, benign or malignant phenotypes were assigned. This assignment was then transmitted to the causative mutations, classifying mutations as benign or malignant (Maron 2002). However, this was contrasted by the finding that the severity of the disease can vary even between patients with the same mutation (Maron 2002; Maron et al. 2012). Therefore additional environmental and genetic factors must influence the disease phenotype (Lopes et al. 2013).

Unequal expression of the two alleles of a gene (allelic imbalance) has been reported for a broad range of genes and tissues and the imbalanced expression of disease associated variants can influence the progression of different diseases (Pastinen 2010). Also for HCM it was shown that the relative abundance of mutant and wildtype mRNA and protein varies between the HCM-mutations (Di Domenico et al. 2012; Helms et al. 2014; Tripathi et al. 2011; Witjas-Paalberends et al. 2013) and the ratio of mutant/wildtype β-MyHC seems to be associated with disease severity (Jiang et al. 2013; Tripathi et al. 2011). In addition to this tissue-level allelic imbalance, we have shown recently that the relative fraction of mutant vs. wildtype mRNA is also variable from cell to cell in the myocardium of HCM-patients (Kraft et al. 2016). We have evidence that this cell-to-cell allelic imbalance is caused by an independent and stochastic ON- and OFF-switching of the mutant and the wildtype *MYH7* allele in each individual cell. In principle, such a stochastic expression should result in nearly equal fractions of mRNA from both alleles at the tissue level. Yet, on average we found the same deviation from a 1:1 relation of mutant and wildtype mRNA for all analyzed single cells as it was determined at the tissue level for the same patients (Kraft et al. 2016; Tripathi et al. 2011). Therefore, factors additional to the stochastic ON- and OFF-switch of the alleles must induce the allelic imbalance at the tissue level. The regulatory mechanisms of allelic expression imbalance encompass variants in different *cis*-regulatory elements such as RNA-stability and turnover, transcription factor binding or splicing regulators but also epigenetic regulators such as DNA methylation (Bjornsson et al. 2008; Milani et al. 2009). Therefore, the allelic imbalance of the *MYH7* gene in HCM may either be affected by intrinsic sequence variations in regulatory regions of the HCM-associated alleles or be directly altered by the mutations.

We hypothesize that if not only the HCM-mutation but also intrinsic, non-HCM-related expression regulating factors on the alleles hold responsible for the *MYH7* allelic expression, allelic imbalance will also be detected in non-HCM controls. To address this question we examined the relative expression of the *MYH7* alleles based on single base substitutions in 11 non-HCM donors and in ten HCM-patients with heterozygous mutations in the *MYH7*-gene.

## Materials and methods

### RNA extraction and RT-qPCR

Muscle tissue of *Musculus soleus* and myocardial tissues were flash frozen directly after excision and stored under liquid nitrogen. RNA was extracted using the PeqGold Total RNA Kit (PeqLab, Erlangen, Germany) according to the supplier’s instructions. Total RNA was subjected to cDNA-synthesis using 1× reaction buffer, 0.125 mM dNTPs each, 0.4 µM *MYH7* specific primers (Table 1), 1 U/µl RNase inhibitor (RiboSafe, Bioline, Luckenwalde, Germany), and 5 U/µl reverse transcriptase (Tetro RT, Bioline) and 1 µl RNA for 1 h at 42 °C. Unless stated otherwise in Table 1, for amplification 1 µl cDNA was mixed with 1x reaction buffer, 0.5 mM MgCl_2_, 0.2 mM of each dNTP, 0.2 µM of both forward and reverse Primers (Table 1), and 0.04 U/µl HotStarTaq (Qiagen, Hilden, Germany). Initial activation was performed for 15 min at 95 °C. Subsequently 45 cycles were applied with 95 °C for 30 s, 64 °C for 30 s, and 72 °C for 30 s. The final elongation was performed at 72 °C for 2 min. To minimize heteroduplexes a reconditioning PCR was performed. 2.5 µl PCR product were transferred to a final volume of 25 µl respective PCR reaction mix, and the respective PCR protocol was run for three successive cycles (Thompson et al. 2002).

**Table 1:** Oligonucleotides and peptides used for allele specific quantification assays

Variant	cDNA-primer	Primer forward	Primer reverse	Buffer	Annealing (°C)	endo-nuclease	Fragments WT	Fragments Mut	Peptide wildtype native	Peptide wildtype isotope	Peptide mutant native	Peptide mutant isotope
A200V	GCCTTGCCTTTGCCCTTCTCA	AGGGTCATCCAGTACTTTGCTGTTATTAC	ATGTCTGCAGATGCCAACTTTCCT	Standard	64	*Hpy*188I	103; **154**	27; 103; **127**	NH2-VIQYFAVIAAIGDR-COOH	NH2-VIQYF*(9C13 + N15)AVIAAIGDR-COOH	NH2-VIQYFAVIAVIGDR-COOH	NH2-VIQYF*(9C13 + N15)AVIAVIGDR-COOH
R453C	GCCTTGCCTTTGCCCTTCTCA	GTTCAACTGGATGGTGACGCGCAT	TTGGCCAGTGCCCCAGTGGCATATAT	Standard	68	*Nla*III	48; **175**	48; 50;**120**				
V606M	TGCCAGGTTGTCTTGTTCCG	TCCCCAAGGCCACCGACATGACCTTCAAGGCCAAGC	CTTTGCCCTTCTCAATAGGCGCATCAG	Standard	68	*Nla*III	20; **268**	20; 88; **180**				
G716R	TGCCAGGTTGTCTTGTTCCG	GTGCTGGAGGGCATCCGCATCT	CCATTCTGGCGAGCACACCT	2,5 mM MgCl_2_	64	*Bsl*I	54; 115; **138**	115; **192**	NH2-GFPNRILYGDFRQRYRILNPAAIPEGQFIDSRK-COOH	NH2-GFPNRIL*(6C13 + N15)YGDFRQRYRIL*(6C13 + N15)NPAAIPEGQFIDSRK-COOH	NH2-GFPNRILYRDFRQRYRILNPAAIPEGQFIDSRK-COOH	NH2-GFPNRIL*(6C13 + N15)YRDFRQRYRIL*(6C13 + N15)NPAAIPEGQFIDSRK-COOH
R719W	TGCCAGGTTGTCTTGTTCCG	ACGAAGTCTCCAGGCGTGATGGACAACC	CTTTTTGTACTCCATTCTGGCGAGCACA	2,5 mM MgCl_2_	68	*Msp*I	109; 123; **146**	109; **269**				
G741R	TGCCAGGTTGTCTTGTTCCG	ATCCCTGAGGGACAGTTCATTGATAGCAGGACG	CTTTTTGTACTCCATTCTGGCGAGCACA	Standard	67	*Bst*UI	51; 171	33; 51; 138	NH2-DSRKGAEKLLSSL-COO3	NH2-DSRKGAEKL*(6C13 + N15)LSSL-COO3	NH2-DSRKRAEKLLSSL-COO3	NH2-DSRKRAEKL*(6C13 + N15)LSSL-COO3
D752N	TGCCAGGTTGTCTTGTTCCG	GGACAGTTCATTGATAGCAGGAAGGGGGCA	CTCGGGACTGGGCCTGGATACGCGTGATCA	2 mM MgCl_2_	68	*Bcl*I	29; 57; 98	29; 155				
T1377M	TCATGGATAGTCTTTCCGCTGGA	GCGTCCTTTCCAAGGCCAACT	AGTTCCTCTGCTTCTTGTCCAGG		65	*Bcc*I	67; **203**	41; 67; **156**				
T63T	GGTTCTGCTGCATCACCTG	CGTGCCTGATGACAAACAGG	GGAGTTGGTGAGTGACAGG	3 mM MgCl_2_	68	*Dde*I	125; **294**	75. 125, **219**				
I989I	GATGCGTGCCTGAAGCTCCTT	CTCAAAAGGGACTCGATGAT	AAAAAAAAAAAAATTCATCCTCAATCATTGC	2 mM MgCl_2_	58	*BsrD*I	24; 116; **318**	24; **434**				
A1702A	TGTCCTCCTCCGTCTGGTAG	CCAACGACGACCTGGCGGAG	CTCAGCATTCCTGCACTCCT	Additional 1 mM MgCl_2_ and 4% DMSO	58	*Eci*I	29; 101; **139**	29; **240**				

### Allele specific restriction digest

12.5 µl of the reconditioned PCR products were treated with respective restriction enzymes (Table 1) in a final volume of 15 µl for at least 3 h to yield the allele specific fragments (Table 1). The fragments were separated on 3% sieving agarose gels stained with ethidiumbromide and mutant vs. wildtype *MYH7* mRNA was quantified as described previously in detail (Tripathi et al. 2011). In brief, the restriction fragments were analysed densitometrically using the TotalLab (Newcastle upon Tyne, Great Britain) and Origin (OriginLab, Northampton, MA, USA) software, yielding the integrated optical density (IOD) of each band. The IOD was normalized against the number of base pairs. The fraction of mutant per wildtype *MYH7* mRNA was calculated from the IOD/bp values of the respective bands.

### Relative quantification of mutant and wildtype myosin

The quantification of mutant and wildtype β-MyHC protein was performed as described previously in detail (Becker et al. 2007). In brief, for each mutation a specific set of isotope labelled peptides (Table 1) was spiked in equal quantities to extracted myosin from tissue samples of the HCM-patients. The mixture was digested using trypsin (A200V), Lys-C (G716R) or Asp-N (G741R) and subjected to LC/ESI-based analysis of the ratio of WT- and mutant-specific peptides in the samples. The isotope labelled peptides were used as internal standards for the quantification to correct for sequence specific ionization. The assays were established using mutant and wildtype specific synthetic peptides (Table 1).

### Statistics

For statistical analysis of the deviation from the 50:50 ratio, we used one way ANOVA test. We compared the fractions of all quantification experiments per mutation or variant, respectively, with the theoretically expected 50%. Analysis was performed using the GraphPad Prism software, significance was assigned for p < 0.0001.

### Ethics statement

Informed consent was obtained from all individuals according to approved Ethics Committee protocols of the institutions involved. The study was approved by the Ethics Committee of Hannover Medical School (no. 2276-2014). The investigations conformed to the principles of the Declaration of Helsinki (1997).

## Results

### Patients and non-HCM donor genetics

We analyzed the allelic expression of the *MYH7* gene in 21 individuals based on single base substitutions (Table 2). We analyzed flash frozen tissue from slow skeletal muscle (*M. soleus*) from one male HCM-patient with the mutation R453C and one male and two female patients with the mutation G741R. In addition, left ventricular/interventricular septum tissue from one HCM-patient each with the mutation A200V, V606M, G716R, R719W, D752N, and T1377M was analyzed. Slow skeletal muscle fibers express mainly the β-myosin isoform and as we have shown previously, the allelic imbalance of the *MYH7* gene is comparable in slow skeletal muscle and heart tissue (Tripathi et al. 2011). The three patients with the mutation G741R were related, however showed distinct progression of HCM at biopsy ranging from asymptomatic (G741R-1, age at biopsy 45), dyspnea on effort (G741R-3, age at biopsy 39) to severe heart failure (G741R-2, age at biopsy 55). Exemplary analysis revealed that the patients A200V, R453C, G716R, G741R-1, G741R-2 and G741R-3 showed no variants in the in the 5′ and 3′-UTR. The patients G741R-1, G741R-2 and G741R-3 were further analyzed for SNPs in the promotor region and also showed no variants.

**Table 2:** Relative expression of the *MYH7* alleles in HCM-patients and non-HCM donors

Variant	Individual	Gender	Tissue	Fraction of mutant allele (mean ± SEM)	
				mRNA	Protein
A200V		Female	Septum	48.3 ± 1.9	49.1 ± 0.9
R453C		Male	Soleus	37.0 ± 1.0*	
V606M		Male	Septum	37.5 ± 0.8*	
G716R		Male	Left ventricle	88.6 ± 0.2*	29.9 ± 0.9*
R719W		Male	Left ventricle	52.2 ± 0.4	
G741R	1	Male	Soleus	18.8 ± 0.4*	21.4 ± 0.4*
	2	Female	Soleus	49.4 ± 1.0	26.9 ± 0.6*
	3	Female	Soleus	44.8 ± 2.9	26.7 ± 0.6*
D752N		Male	Septum	7.9 ± 0.6*	
T1377M		Female	Septum	52.3 ± 1.1	
T63T	1	Male	Left ventricle	51.1 ± 0.33	
	2	Female	Left ventricle	49.2 ± 0.97	
	3	Male	Left ventricle	5.5 ± 0.15*	
I989I	1	Female	Left ventricle	51.2 ± 0.04	
	2	Female	Left ventricle	51.3 ± 0.1	
	3	Female	Left ventricle	53.2 ± 0.1	
	4	Male	Left ventricle	52.6 ± 0.1	
A1702A	1	Female	Left ventricle	60.2 ± 0.2*	
	2	Male	Left ventricle	60.3 ± 0.7*	
	3	Female	Left ventricle	58.1 ± 0.2*	
	4	Male	Left ventricle	50.1 ± 0.3	

*Significant deviation from 50% of equal allelic expression (p > 0.0001)

The *MYH7*-variants R453C and V606M (Watkins et al. 1992), G716R and R719W (Anan et al. 1994), and G741R (Fananapazir et al. 1993) have been previously described as disease causing and are listed as “pathogenic” in the ClinVar database. In addition, they are reported with a very low allele frequency or are even not present in the Exac or GnomAD databases and at least three out of four in silico prediction softwares report the variants as pathogenic (Table 3). Therefore, these HCM-variants strongly fulfill the criteria of pathogenicity according to the ACMG guidelines (Richards et al. 2015). The mutation T1377M is listed as “likely pathogenic” in ClinVar, it has been described previously as HCM-mutation (Helms et al. 2014) and it is highly seldom in the reference population as determined by the GnomAD allele frequency of 4.06 × 10^− 6^. In addition, *in silico* prediction of pathogenicity by two out of four independent software tools suggests a deleterious effect on the mutant protein (Table 3). According to the ACMG guidelines (Richards et al. 2015) this indicates strongly to a pathogenic effect of the T1377M-variant. The variants A200V and D752N are not listed in ClinVar. However, the variant alleles are not present in the Exac/GnomAD database and both variants are predicted to be deleterious for protein function by the three in silico analysis tools. In addition, at position 200 another pathogenic variant has been determined (A200T) (Fujino et al. 2013). Taken together, the ACMG guidelines (Richards et al. 2015) strongly support a pathogenic effect also of these mutations (Table 3). Therefore, we assume that all *MYH7*-variants in the HCM patients as pathogenic under the light of current evidence.

**Table 3:** Pathogenicity of *MYH7*-variants of the HCM-patients and donors

Variant	Exac/GnomAD allele frequency	In silico predictions of pathogenicity				ClinVar classification	ACMG classification^e^	Initial reference for pathogenic variant
		Mutation taster^a^	PolyPhen2^b^	PhD-SNP^c^	PANTHER^d^			
A200V	Not present	Disease causing	Probably damaging	Disease	Neutral	No data	Pathogenic (PS1 + PM2 + PP3)	Pathogenic variant at same amino acid: Fujino et al. (2013)
R453C	Not present	Disease causing	Probably damaging	Disease	Disease	Pathogenic	Pathogenic (known disease causing, PS3 + PM2 + PP3)	Watkins et al. (1992)
V606M	3.230 × 10^−5^	Disease causing	Probably damaging	Neutral	Disease	Pathogenic	Pathogenic (known disease causing + PS3 + PM2 + PP3)	Watkins et al. (1992)
G716R	Not present	Disease causing	Probably damaging	Disease	Disease	Pathogenic	Pathogenic (known disease causing PS3 + PM2 + PP3)	Anan et al. (1994)
R719W	3.231 × 10^−5^	Disease causing	Probably damaging	Disease	Disease	Pathogenic	Pathogenic (known disease causing PS3 + PM2 + PP3)	Anan et al. (1994)
G741R	3.232 × 10^−5^	Disease causing	Probably damaging	Disease	Neutral	Pathogenic	Pathogenic (known disease causing PS3 + PM2 + PP3)	Fananapazir et al. (1993)
D752N	Not present	Disease causing	Probably damaging	Disease	Neutral	No data	Pathogenic (PS2 + PM2 + PP3)	Not described previously
T1377M	4.06 × 10^−6^	Disease causing	Probably damaging	Neutral	Neutral	Likely pathogenic	Pathogenic (known disease causing, PS2 + PM2 + PP3)	Helms et al. (2014)
T63T	0.47960	Polymorphism	Not applicable	Not applicable	Not applicable	Benign	Benign (BA1)	
I989I	0.32119	Polymorphism	Not applicable	Not applicable	Not applicable	Benign	Benign (BA1)	
A1702A	0.10702	Polymorphism	Not applicable	Not applicable	Not applicable	Benign	Benign (BA1)	

^a^Schwarz et al. (2014)

^b^Adzhubei et al. (2013)

^c^Capriotti et al. (2006)

^d^Mi et al. (2016)

^e^Richards et al. (2015)

In addition, interventricular septum samples from non-HCM donors were analyzed for single nucleotide polymorphisms (SNPs) in the *MYH7* gene. We identified three donors heterozygous for the SNP T63T (rs2069540), four donors heterozygous for the SNP I928I (rs7157716) and four donors heterozygous for the SNP A1702A (rs3729830). Under the light of current evidence, the variants are not pathogenic (Table 3). To our knowledge, the donors were not related to each other. In total five male and six female donors were analyzed.

### Allelic balance and imbalance of the *MYH7* gene in HCM-patients

We have shown previously, that the mutant and wildtype *MYH7* alleles are expressed imbalanced at the tissue level in HCM-patients with different missense mutations in this gene. To examine whether this phenomenon can be detected for a broader range of *MYH7*-mutations, we determined the ratio of mutant vs. wildtype transcript in samples of slow skeletal muscle tissue and cardiac tissue from HCM-patients with eight different *MYH7*-missense mutations.

**Fig. 1:**
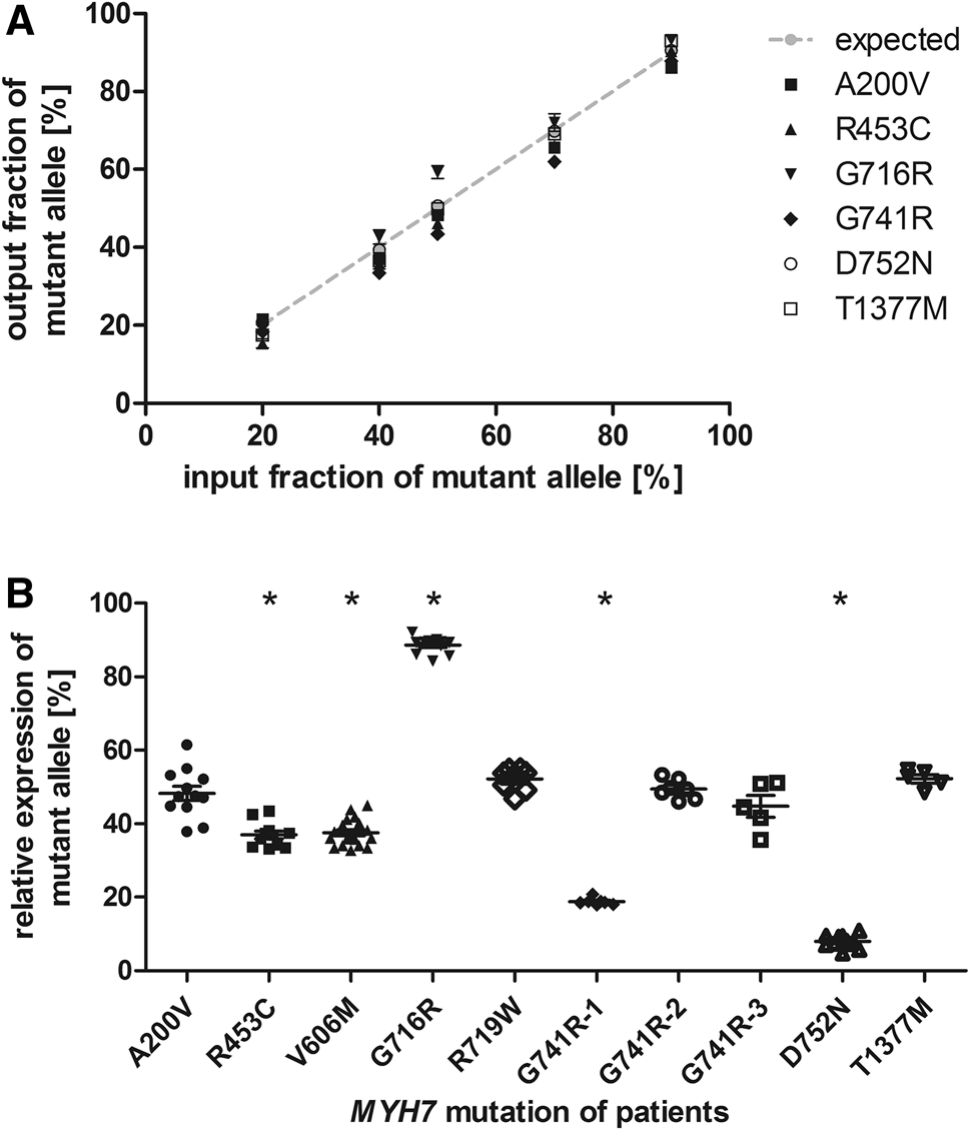
Relative quantification of mutant and wildtype *MYH7*-mRNA in HCM-patients. **a** Defined mixtures of synthetic plasmids encoding for the wildtype and the respective mutated sequences were amplified by quantitative PCR and quantified densitometrically from allele specific restriction digests. Quantifications were performed in at least 4 independent experiments. The expected ratios are indicated by the grey line. **b** Relative quantification of mutant and wildtype *MYH7-*mRNA in *M. soleus* or myocardial tissue from HCM-patients. Quantification was performed on RNA extracted from at least two pieces of tissue at least in duplicate analysis. Each dot represents a single quantification analysis. The asterisks indicate significant deviation from 50% (one way ANOVA; p < 0.0001)

The analyses were designed as shown previously for HCM-associated *MYH7-*mutations (Tripathi et al. 2011). In brief, the PCR amplicons contained a restriction site that was either generated or disrupted by the respective base substitution. The region of interest was PCR-amplified, reconditioning PCR was performed to eliminate potential heteroduplexes originating from the end point PCR and subsequently subjected to restriction digest. The allele specific restriction fragments were separated by agarose gel electrophoresis, quantified densitometrically and the respective ratios of each allele were calculated. Each analysis was optimized and finally validated using defined mixtures of recombinant plasmids encoding either for the wildtype or the mutant PCR amplicon. Using the finalized protocols, we relatively quantified both alleles in the standard plasmid mixtures for ratios between 10:90 and 80:20 in at least three independent experiments (Fig. 1A). For each patient 2–4 independent RNA extractions were performed and used for at least two cDNA-syntheses. The expression of the mutant allele was quantified from each cDNA at least in duplicates and statistically compared to a theoretical value of 50% reflecting balanced allelic expression (one way ANOVA) (Fig. 1b).

The mutations R435C, V606M, and D752N showed a significantly reduced expression of the mutated allele of on average 37.0, 37.5 and 7.9%, respectively (Fig. 1b). In the patients with the mutations A200V, R719W, and T1377M both alleles were expressed at a similar ratio of 48.3, 52.2, and 52.3%, respectively. The mutation G716R showed a high expression of the mutant allele with 88.6% (Table 2).

Interestingly, in skeletal muscle tissue from three related patients with the mutation G741R we found a varying expression pattern. Whereas one patient (G741R-1) showed a significantly reduced expression of the mutated allele of 18.8%, both other patients (G741R-2 and G741R-3) showed equal expression of both alleles of 45.6 and 46.6% of the mutant allele (Fig. 1b; Table 2). To test for differences between the G741R patients we compared SNPs in the promotor region and in exons and flanking intron sequences. No variant was identified in the promotor region or the 3′ UTR. However, the patients showed different exonic SNPs (Table 4).

**Table 4:** Variants detected upon analysis of the *MYH7* promoter, 5′-UTR, 3′-UTR, exons, and flanking intron sequences

Patient	Location	Web Ref.	HGVS nomenclature	Effect	Genotype
G741-1	Exon	rs2069540	c.189C>T	SNP	Homozygous
	Exon		c.2221G>C	Mutation	Heterozygous
	Exon	rs3729830	c.5106G>A	SNP	Heterozygous
G741R-2	Exon	rs2069540	c.189C>T	SNP	Heterozygous
	Exon		c.1095G>A	SNP	Heterozygous
	Exon		c.2221G>C	Mutation	Heterozygous
	Exon	rs7157716	c.2967T>C	SNP	Heterozygous
G741R-3	Exon	rs2069540	c.189C>T	SNP	Homozygous
	Exon		c.2221G>C	Mutation	Heterozygous

However, at the protein level the difference between patients with the mutation G741R was no longer detectable. The myocardial tissue of the three patients contained 21.4, 26.9, and 26.7%, respectively, of the mutant β-MyHC protein (Table 2). Also the patient with mutation G716R showed different levels of mutant mRNA and protein. At the mRNA level the mutant allele was predominant with 89%, while at the protein level only 29.9% mutant myosin was found. The patient with mutation A200V in contrast showed comparable levels of mutant mRNA and protein.

### Allelic imbalance of the *MYH7* gene in non-HCM individuals

To test whether the *MYH7* alleles are generally expressed imbalanced and that this is not specific for HCM patients, we analyzed cardiac tissue from non-HCM control individuals. Three different single nucleotide polymorphisms within the *MYH7* gene were used for allelic discrimination.

**Fig. 2:**
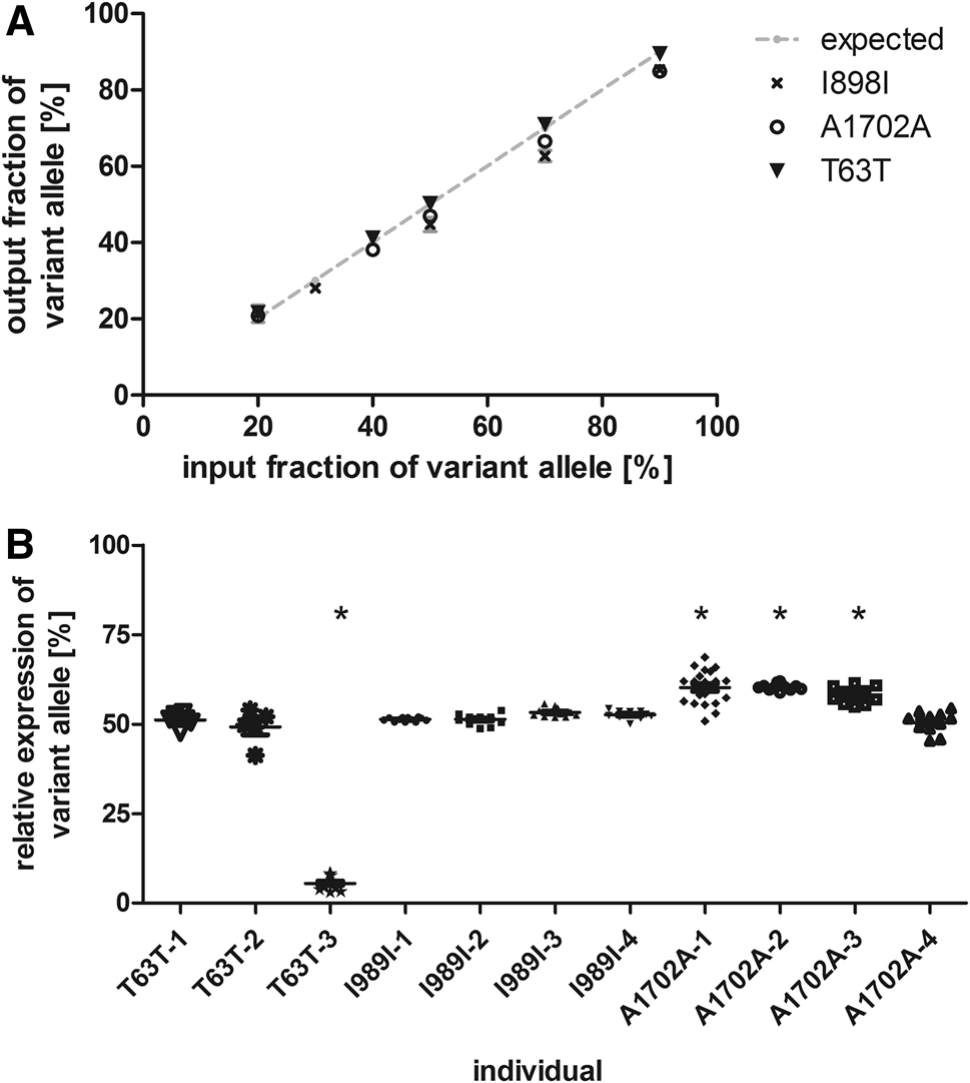
Relative quantification of the *MYH7* alleles in myocardium of non-HCM control individuals using heterozygous single nucleotide polymorphisms. **a** Defined mixtures of synthetic plasmids encoding for the wildtype and T63T (triangle) I989I (cross) or A1702A (open circle) variant, respectively were amplified by quantitative PCR and quantified densitometrically from allele specific restriction digests. Quantifications were performed in at least four independent experiments. The expected ratios are indicated by the grey line. **b** Relative quantification of *MYH7*-mRNA in myocardial tissue from 11 non-HCM control individuals carrying the variants T636T, I989I, and A1702A, respectively. Quantification was performed on RNA extracted from at least two pieces of tissue at least in duplicate analysis. Each dot represents a single quantification analysis. The asterisks indicate significant deviation from 50% (one way ANOVA; p < 0.0001)

Specific qPCR and restriction digest analyses were established for each SNP and validated using standard plasmid mixtures as described for the HCM-mutations (Fig. 2a). For each individual, three independent RNA extractions were performed and analyzed as described for the HCM-patients. Most interestingly, we determined a statistically significant deviation from the 50:50 allelic expression ratio in four out of 11 non-HCM donors (Fig. 2b). For three control individuals we determined up to 60% of the variant allele, and one control individual showed a low fraction of the variant allele of on average 5.5% (Table 2). Therefore, the allelic expression ranges from equal to almost exclusively one allele. Nevertheless, both alleles were expressed in all individuals.

## Discussion

### Allelic imbalance is a general feature of *MYH7* mRNA expression

Several studies on HCM-patients have shown unequal expression of the mutant and wildtype alleles of β-myosin (*MYH7*) (Di Domenico et al. 2012; Nier et al. 1999; Tripathi et al. 2011; Witjas-Paalberends et al. 2013) and myosin binding protein (*MYBPC3*) (Helms et al. 2014) at mRNA and protein level. Also balanced allelic expression of the mutant and wildtype mRNA associated with imbalanced expression of mutant and wildtype protein have been reported (Helms et al. 2014). Clinically, the severity of HCM has been linked to the fraction of mutant protein (Jiang et al. 2013; Tripathi et al. 2011) and to the degree of imbalance (Helms et al. 2014). In line with these findings our present study shows *MYH7-*allelic expression imbalance and balance in the HCM-patients, both at the mRNA and at the protein level. The allelic imbalance encompasses a broad range, from an almost exclusive expression of either the mutant or the wildtype allele, respectively, to a balanced expression of both alleles. However, allelic expression imbalance has also been shown for many different genes under healthy conditions (Jeffries et al. 2016). In accordance, also non-HCM control individuals showed imbalanced expression of the *MYH7*-alleles. Next to only slight deviations from the 50:50 ratio also a marked imbalance was detected. Our findings contradict the assumption that *MYH7*-missense mutations cannot be expressed imbalanced in HCM-patients at the mRNA or protein level (Helms et al. 2014). The authors assume, that only premature termination codons would cause allelic imbalance. In contrast, our study shows, in line with findings from other groups (Heap et al. 2009; McDaniell et al. 2010; Serre et al. 2008), that allelic imbalance is a common mechanism of gene expression; also under non-disease associated conditions. Therefore, our findings provide evidence that a general variation in *MYH7*-allelic expression exists and importantly, may also underlie at least part of the allelic imbalance experienced in HCM-patients.

At the mRNA-level, a marked allelic imbalance was only found in male individuals. However, in previous studies we have also determined ratios of 70/30 in a female patient (H29, Tripathi et al. 2011). Therefore, we assume that our cohort is not representative with regard to gender diversity. In addition, at the protein level both, male and female HCM-patients show significant allelic imbalance.

It should be noted that we used the different SNPs in the non-HCM individuals only as indicators for the differential expression of the *MYH7-*alleles. These SNPs are very common (Table 3) and are also found in some of the HCM-patients (Table 4). Since the allele specific expression levels vary largely between individuals with the same SNPs we do not assume that the examined SNPs dominate allelic imbalance establishment. However, it seems likely that at least some SNPs may contribute significantly to regulation of the allelic expression of the *MYH7-*alleles.

Our results from donor individuals show for the first time, that allelic imbalance is an intrinsic, non-HCM-dependent feature of *MYH7*-gene expression. Under healthy conditions—without disease causing mutations—the imbalanced allelic expression of genes will have no further effect on functional parameters. However, altered expression of variants that affect protein function or increase disease susceptibility may exhibit severe effects on the metabolism, e.g. cardiomyocyte and thus cardiac function. Finally, this may increase the severity or occurrence of the respective disorder as shown for multiple sclerosis (Keshari et al. 2016), autoimmune diseases (Ge et al. 2009), cancer (Chen et al. 2008; Wang et al. 2016) and HCM (Di Domenico et al. 2012; Helms et al. 2014; Tripathi et al. 2011). And vice-versa, in a mouse model of HCM, the targeted knock-down of the mutant allele has been shown to improve HCM-phenotype (Jiang et al. 2013). Therefore, next to the direct “poisoning” effect of the *MYH7*-mutations on β-MyHC function, the unequal expression of mutated and wildtype alleles may provide an essential player in HCM development (Brenner et al. 2014). Our results indicate that in patients with HCM-mutations, the intrinsic *MYH7*-allelic imbalance may be one additional factor that affects disease progression.

### Mutation dependent and independent mechanisms regulate unequal allelic expression of *MYH7*

In a previous study we have shown that the *MYH7* alleles are expressed at different ratios in neighboring cells in the myocardium, causing a functional imbalance between these cells that may finally trigger the progression of myofibrillar and myocardial disarray and hypertrophy (Kraft et al. 2016). We assume that this cell-to-cell allelic imbalance is caused by independent, stochastic ON- and OFF-switching of each *MYH7*-allele, so-called bursts-of-transcription. However, the stochastic expression pattern should result in equal levels of each allele averaged over the multitude of cells analyzed in our tissue samples. Nevertheless, the average of all single cells in patients with the mutation R723G show a comparable level of allelic imbalance as determined at the tissue level (Kraft et al. 2016). In consequence, additional mechanisms must cause an average drift of the allelic expression towards one allele. Our present study did not address the exact mechanisms of allelic expression establishment. However, our findings allow to draw some conclusions that may provide deeper insights into such potential mechanisms.

Exemplary analysis of the promotor region in three patients and the 5′- and the 3′-UTR in 6 patients revealed no variant. Variants in these regions can affect mRNA- and protein levels by altering transcription factor- or miRNA-binding, respectively. Based on the absence of variants in the analyzed patients with and without allelic imbalanced expression, we assume no influence on allelic expression regulation in these cases. Nevertheless, in other patients imbalance may be induced by variants in these regions.

The observation of unequal expression of the two *MYH7*-alleles in non-HCM control individuals suggests that allelic imbalance of *MYH7* is an intrinsic feature of this gene, which may be modulated by HCM-associated missense mutations. Interestingly, in most cases the relative fraction of mutant alleles seems to be comparable between patients with identical mutations as we have shown previously for related patients (I736T) and unrelated patients with presumably a common founder mutation (R723G) (Enjuto et al. 2000; Tripathi et al. 2011). Even though this may be explained by identical inherited - but mutation independent - regulatory motifs, we have hypothesized that also a mutation induced alteration of the mRNA turnover may cause this imbalance (Tripathi et al. 2011). In line with this hypothesis, here we found comparable allelic imbalance in three patients unrelated to patients from previous studies: the patient with mutation V606M had 38% mutated mRNA in myocardial tissue, which is similar to the previously determined 28% mutated mRNA in *M. soleus* (Tripathi et al. 2011). In addition, patients with the mutations R719W and T1377M, respectively, show only a moderate increased expression of the mutant allele or a balanced expression of both alleles in this and previous studies (Helms et al. 2014; Tripathi et al. 2011). This supports the assumption that the mutations may also directly influence the allelic expression (Tripathi et al. 2011). E.g. we have preliminary evidence that the mutation R723G alters mRNA secondary structure (unpublished data), indicating that mutations can influence the mRNA stability. In addition point mutations can act as a *cis*-regulatory variant for methylation, alter the affinity of RNA-binding proteins that affect recruitment of DNA-methylation complexes or histone modifications (Zhang et al. 2009).

Next to mutation associated regulators, our results indicate that also mutation independent factors influence the allelic expression. One of the three patients with the mutation G741R has a significantly lower fraction of the mutant mRNA compared to the others. Analysis of the promotor and 3′-UTR (Table 4) did not reveal any difference between the patients, indicating that here either intragenic or epigenetic factors may also affect the allelic expression of *MYH7*. Regulation of allelic expression biases have been assumed to be genetically determined (Pastinen 2010) and also epigenetically regulated (Tycko 2010). Especially the allele specific methylation (ASM) and allele specific transcription factor binding (ASTF) have been determined as potent *cis-*acting regulators of allelic imbalance (Jeffries et al. 2016; Nakaoka et al. 2016; Tycko 2010). The expression of the *MYH7* gene is regulated by DNMT3a dependent methylation of the promotor (Fang et al. 2016). Therefore, allele specific levels of methylation of the *MYH7* promotor may also provide one mechanism for the establishment of allelic imbalance irrespective of the HCM mutations.

Taken together, our data indicate that the *MYH7*-alleles can be expressed imbalanced in healthy individuals, without causing cardiac pathologies. In patients with HCM-mutations, this intrinsic imbalance may then be one factor that determines disease progression. However, most patients with identical mutations show comparable levels of allelic imbalance. Therefore, in addition to the intrinsic allelic imbalance, also mutation-associated regulators may determine the allelic expression.

### Transcriptional and post-transcriptional mechanisms induce allelic imbalance of mutant and wildtype β-MyHC at the protein level

In six out of six HCM-patients from a previous study (Tripathi et al. 2011) and in two out of five mutations in this study the allelic imbalance at the protein level is comparable to the mRNA allelic imbalance. This in accordance with the assumption that “Protein Levels at Steady State Are Primarily Determined by mRNA Levels” (Liu et al. 2016). Therefore, we assume that in most cases the *MYH7*-allelic imbalance is induced prior to translation of the *MYH7*-gene and thus mRNA imbalance is paralleled by protein level imbalance.

Most interestingly, the missense mutations G741R and G716R show lower fractions of mutated protein as compared to the respective mutated mRNA fractions. The examined mutations are missense mutations that do not cause frame shifts. Therefore, mechanisms such as nonsense mediated decay are highly unlikely (Helms et al. 2014). The disparity of mRNA and protein levels can be linked to different mechanisms as reviewed in (Maier et al. 2009): first, the RNA secondary structure can alter the translation efficacy. As stated above, we have preliminary evidence, that HCM-mutations may alter the secondary structure of the mRNA. Future analyses will address a possible effect of these specific mutations on the secondary structure. Second, regulatory RNAs such as miRNAs may inhibit mRNA translation. However, analysis of the 5′- and the 3′-UTR of the patients revealed no variant, therefore an aberrant miRNA-binding seems rather unlikely. Third, the ribosomal density can regulate the translation efficiency. Determinants of ribosomal density are located in the 5′-UTR (Dvir et al. 2013), which contains no variants in the analyzed patients. Therefore, the ribosomal density will most likely not affect the protein levels. Forth, a reduced protein half-life can lead to reduced levels of protein. The HCM-mutations G716R and G741R could lead to proteins that are degraded faster than the wildtype proteins. This would lead to the observed decreased mutant:wildtype ratio at the protein as compared to the mRNA level. Future experiments will have to show, whether the half-life of the β-MyHC protein is shortened by the mutations G716R and G741R. Last but not least, alternative splicing resulting in non-functional proteins may have caused the reduced mutant protein levels as compared to the mRNA. For myosin binding protein C (*MYBPC3*) mutations, differential splicing of wildtype and mutant mRNA is an important mechanism that affects protein level imbalance (Helms et al. 2014). For *MYH7* mutations splice effects have not been experimentally determined so far, however, bioinformatically predicted (Tripathi et al. 2011). Also for the base substitution c.2146G>A leading to the mutation G716R the generation of an alternative splice site, however with a comparable activity score as the original splice site, is predicted (Desmet et al. 2009). Nevertheless, differential splicing may act as one mechanism underlying protein level allelic imbalance. Most interestingly, the three G741R patients with different fractions of mutant mRNA showed comparable fractions of mutant and wildtype protein for all three patients. Here a post-transcriptional mechanism seems to establish a constant mutant protein ratio, irrespective of the initial fraction of mutant mRNA. Further studies will have to clarify the post-transcriptional mechanisms that affect the expression of mutant and wildtype β-MyHC.

## Conclusion

Our study provides evidence that the *MYH7* alleles can be expressed balanced and highly imbalanced at the mRNA and at the protein level in both, HCM-patients and non-HCM controls. In non-HCM control individuals, the imbalanced expression will have no physiological impact. However, in HCM-patients one allele encodes for a functionally altered protein of the contractile apparatus. The influence of this alteration may increase with an increasing fraction of mutant protein. Our results suggest that diverse mechanisms must exist for the establishment of allelic imbalance not only between patients with different mutations but also between patients sharing identical HCM-mutations.
